# Increased reward value of non-social stimuli in children and adolescents with autism

**DOI:** 10.3389/fpsyg.2015.01026

**Published:** 2015-07-22

**Authors:** Karli K. Watson, Stephanie Miller, Eleanor Hannah, Megan Kovac, Cara R. Damiano, Antoinette Sabatino-DiCrisco, Lauren Turner-Brown, Noah J. Sasson, Michael L. Platt, Gabriel S. Dichter

**Affiliations:** ^1^Institute for Cognitive Science, University of Colorado at Boulder, Boulder, CO, USA; ^2^Carolina Institute for Developmental Disabilities, Chapel Hill, NC, USA; ^3^Geisinger Autism Center, Lewisburg, PA, USA; ^4^TEACCH Autism Program, UNC School of Medicine, The University of North Carolina at Chapel Hill, Carrboro, NC, USA; ^5^School of Behavioral and Brain Sciences, University of Texas at Dallas, Dallas, TX, USA; ^6^Department of Neuroscience, Perelman School of Medicine, University of Pennsylvania, Philadelphia, PA, USA; ^7^Department of Psychiatry, University of North Carolina at Chapel Hill School of Medicine, Chapel Hill, NC, USA

**Keywords:** autism, social, pay-per-view, reward, restricted interests, behavioral economics

## Abstract

An econometric choice task was used to estimate the implicit reward value of social and non-social stimuli related to restricted interests in children and adolescents with (n = 12) and without (n = 22) autism spectrum disorder (ASD). Mixed effects logistic regression analyses revealed that groups differed in valuation of images related to restricted interests: control children were indifferent to cash payouts to view these images, but children with ASD were willing to receive less cash payout to view these images. Groups did not differ in valuation of social images or non-social images not related to restricted interests. Results highlight that motivational accounts of ASD should also consider the reward value of non-social stimuli related to restricted interests in ASD (Dichter and Adolphs, 2012).

## Introduction

Social impairments are a defining feature of autism spectrum disorder (ASD). This has been demonstrated in the domains of social cognition (e.g., theory of mind), social perception, and social attention (Levy et al., 2009). Recently, there has been increased interest in examining the impact of motivational factors on social functioning in ASD (Dichter and Adolphs, 2012). The social motivation theory of ASD posits that disruptions in social motivation constitute a primary deficit in ASD, ultimately resulting in fewer experiences with social sources of information and decreased social learning (Dawson et al., 2005; Chevallier et al., 2012b). Consistent with this model, very young children with ASD demonstrate decreased orienting to social stimuli (Dawson et al., 1998; Klin et al., 2009), and atypical social orienting has been shown to predict decreased social competence in adolescents with ASD (Klin et al., 2002). There is also evidence that social motivation remains impaired in individuals with ASD despite growth in other areas of cognitive development (Chevallier et al., 2012a), highlighting the significance of motivational impairments to ASD etiology.

Decreased social motivation is likely not the only mechanistic account of the full range of social deficits associated with ASD. For example, some individuals with ASD have social interests and actively seek out social interactions but fail to form friendships due to poor social skills and pragmatic language. This is perhaps not surprising given that ASD is a complex and heterogeneous neurodevelopmental disorder in which no single etiology accounts for the full range of symptom expression (Murdoch and State, 2013). However, even during the first year of life, infants with ASD demonstrate infrequent orienting to their own name and diminished eye contact (Ozonoff et al., 2010; Jones and Klin, 2013), suggesting that decreased social interest is evident in infancy in ASD and may interfere with the development of social cognition in at least a significant proportion of individuals with ASD.

Despite a crucial role for compromised social behavior in ASD, results of behavioral studies designed to test altered social motivation in ASD have been mixed. On one hand, some studies have found diminished responsiveness to social reward. For example, in an incentive delay task, children with ASD responded faster to monetary than social rewards (Demurie et al., 2011), and, unlike typically developing (TD) children, children with ASD did not show significant improvements in a Eriksen Flanker task under a social motivation condition (Geurts et al., 2008). On the other hand, other studies have indicated either no motivational deficits for social rewards, or that motivational deficits in ASD may not be constrained to social rewards. For example, in a go-no task, there was no difference between ASD participants and TD controls when performing for either monetary or social rewards, though ASD individuals made more errors in the unrewarded condition (Pankert et al., 2014). Moreover, children with ASD did not differ from TD children in their willingness to work to view faces, inverted faces, or cars, suggesting no differences in incentive motivation in ASD when viewing either social or non-social stimuli (Ewing et al., 2013). In another study, there were no differences between ASD and control individuals when reporting valence ratings to positive and negative social feedback, and no overall behavioral difference between the two groups when this social feedback was used to incentivize performance on an instrumental probabilistic learning task (Lin et al., 2012). However, in this study individuals with ASD had impaired *learning* relative to TD individuals when playing the task for social, but not monetary rewards (Lin et al., 2012). The latter finding highlights the possibility that, in the context of behavioral paradigms designed to measure altered motivation and valuation, behavioral differences may be subtle and difficult to detect in individuals with ASD.

Notably, some functional imaging studies reveal neural activation differences related to different types of rewards in ASD, even in the absence of behavioral differences. For example, in an incentive delay task performed for social or monetary rewards, there were no interactions between reward type and subject group (ASD vs. TD individuals) on reaction time, though individuals with ASD showed less improved reaction times on rewarded trials overall (Delmonte et al., 2012). Individuals with ASD did, however, exhibit attenuated activity in the dorsal striatum when performing for social, but not monetary rewards (Delmonte et al., 2012).

Functional neuroimaging and EEG studies testing whether altered brain function during motivated behavior in ASD is specific to social stimuli have also revealed mixed findings. Some studies find differences in ASD in response to social, but not non-social stimuli (Delmonte et al., 2012; Stavropoulos and Carver, 2014); some find differences in response to both social and non-social stimuli with larger effect sizes in the case of social stimuli (Scott-Van Zeeland et al., 2010); and some find no differences between social and non-social stimuli (Kohls et al., 2011, 2012). These findings are consistent with emerging evidence that motivational impairments in ASD are not constrained to responses to social stimuli. Functional neuroimaging studies have found atypical activation in canonical social and reward processing brain regions in ASD during the processing of images of a preferred cartoon character (Grelotti et al., 2005), food (Cascio et al., 2012), and monetary rewards (Schmitz et al., 2008; Kohls et al., 2012), suggesting that motivational impairments in ASD may extend beyond impairments processing social reward cues. In children with ASD, reward circuit activity in response to images of restricted interests is preserved even though reward circuit activity is decreased for monetary rewards (Dichter et al., 2012a). Moreover, in an operant task in which participants used an effortful key press sequence to control the display time of images of neutral objects and objects of restricted interest, there was similar behavioral performance across ASD and TD participants, but greater activity in the insula and anterior cingulate cortex in ASD participants in response to images of restricted interest (Cascio et al., 2014). These findings raise the possibility that restricted interests in ASD co-opt resources typically allocated toward social stimuli.

To date, no research has used behavioral econometrics to investigate the implicit reward value of social and non-social stimuli in ASD. In the present investigation, we used an econometric choice task to determine whether children and adolescents with and without ASD showed differences in the implicit reward value of social and non-social stimuli. The econometric choice task was modified from a task previously used to determine social value in non-clinical contexts (Hayden et al., 2007), to evaluate social motivation in individuals with anorexia nervosa (Watson et al., 2010), and to examine social motivation in rhesus macaques (Deaner et al., 2005). The task provides a quantitative measure of the tendency to approach or avoid a particular class of images based on the degree to which they add positive or negative reinforcement to a monetary reward. This approach is particularly appropriate to investigate reward value in ASD given that self-reports of internal states are of questionable validity in ASD (Hill et al., 2004). We examined choices in the context of (1) social stimuli (i.e., images of smiling faces); (2) non-social images related to restricted interests in ASD; and (3) non-social images not related to restricted interests in ASD. Consistent with the social motivation theory of ASD, we predicted that the ASD group would demonstrate decreased valuation of social stimuli. Consistent with prior findings that restricted interest stimuli recruit reward processing brain regions in ASD (Cascio et al., 2012; Dichter et al., 2012a), we predicted that the ASD group would demonstrate increased valuation of only non-social images related to restricted interests in ASD. We further hypothesized that differential valuation of social and non-social restricted interest stimuli would predict individual differences in the severity of ASD symptoms in the ASD sample.

## Materials and Methods

### Participants

Twelve children and adolescents with ASD (75% male; age range: 10.8–18.9) and 22 matched controls (91% male; age range: 9.3–17.3) completed a behavioral econometric choice evaluation task. Participants consented to a protocol approved by the local human investigations committee at UNC-Chapel Hill. Diagnostic groups did not differ in terms of age or intelligence quotient scores [derived from the Kaufman Brief Intelligence Test (KBIT, Kaufman, 1990)], all *p*’s > 0.05 (see Table 1).

**TABLE 1:** **Participant characteristics**.

	**ASD (*n* = 12)**	**Control (*n* = 22)**	***t*(*p*)**
Age	15.3 (2.9)	13.4 (2.5)	1.91 (0.07)
ADOS total score	15.4 (3.5)	–	–
ADOS calibrated severity score^d^	10.3 (4.4)	–	–
SRS^b^			
Awareness	9.7 (3.1)	10.9 (2.6)	1.21 (0.23)
Cognition*	17.5 (4.9)	11.2 (2)	5.28 (<0.0001)
Communication*	26.1 (7.8)	17.1 (2.9)	4.87 (<0.0001)
Mannerisms*	16.1 (5.5)	1.4 (1.4)	11.96 (<0.0001)
Social motivation*	14.3 (4.6)	9.5 (2.0)	4.45 (0.0002)
Total score*	73.7 (9.5)	57.9 (3.2)	6.98 (<0.0001)
RBS-R^c^			
Stereotyped behavior*	3.3 (2.1)	0.1 (0.4)	6.92 (<0.0001)
Self-injurious behavior*	1.3 (1.2)	0 (0.2)	4.58 (<0.0001)
Compulsive behavior*	4.8 (5)	0.3 (0.8)	4.23 (0.0002)
Ritualistic behavior*	4.6 (3.1)	0 (0.2)	6.95 (<0.0001)
Sameness behavior*	6.4 (4)	0.1 (0.3)	7.42 (<0.0001)
Restricted behavior*	2.8 (1.4)	0 (0.2)	9.26 (<0.0001)
Total score*	23.2 (13.1)	0.6 (1.2)	8.09 (<0.0001)
Verbal IQ^a^	109.5 (17.3)	110.8 (11.9)	0.26 (0.79)
Performance IQ^a^	107.7 (16.8)	110.9 (11.4)	0.67 (0.51)
Full scale IQ^a^	110.2 (17.7)	113 (11.5)	0.56 (0.58)

^a^IQ, Intelligence Quotient derived from the Kaufman Brief Intelligence Test (KBIT); ^b^SRS, Social Responsiveness Scale (Constantino and Gruber, 2002); ^c^RBS-R, Repetitive Behavior Scale-Revised (Bodfish et al., 1999); Standardized severity scores on a scale of 1–10 calculated from raw Autism Diagnostic Observation Schedule (ADOS) scores (Gotham et al., 2009; Hus and Lord, 2014). *p < 0.001.

Seven children in the ASD group were on psychotropic medication, including psychostimulants, benzodiazepines, and selective serotonin reuptake inhibitors. All participants with ASD had clinical diagnoses of ASD confirmed through the Autism Diagnostic Observation Schedule-Generic (ADOS-G; Lord et al., 2000) administered by reliable assessors supervised by a licensed clinical psychologist and using standard cutoffs. Because both Module 3 (*n* = 5) and Module 4 (*n* = 7) were used, calibrated severity scores were calculated from raw ADOS scores to obtain dimensional measures of ASD symptom severity (Gotham et al., 2009; Hus and Lord, 2014). Both groups also completed the Social Responsiveness Scale (Constantino and Gruber, 2002), a dimensional measure of overall ASD symptom severity as well as the Repetitive Behavior Scale-Revised (Bodfish et al., 1999), a dimensional measure of repetitive behavior severity in ASD. Control participants scored below the recommended cutoff of 15 on the Social Communication Questionnaire (Mulligan et al., 2009).

### Stimulus Sets

All images were in color. Participants completed three task blocks, each with one of the following stimulus categories.

#### Social Stimuli

Social stimuli consisted of 52 Happy-Direct Gaze images from the NIMH Child Emotional Faces Picture Set (NIMH-ChEFS; Egger et al., 2011), a standardized set of male and female child faces.

#### Non-Social Restricted Interests Stimuli

Although the restricted interests of individuals with ASD are, by definition, idiosyncratic, a standardized image set of 34 images related to common restricted interests in ASD (e.g., trains and electronics) was used to allow all participants to view the same images (images are presented in the Appendix of Dichter et al., 2012a). These images were derived from categories of common restricted interests in ASD (South et al., 2005), have been shown to differentially activate brain reward circuitry in ASD (Dichter et al., 2012a), to elicit great visual attention in children and adults with ASD in eyetracking paradigms (Sasson et al., 2011, 2008), and rated as more pleasing by individuals with ASD (Sasson et al., 2012). These stimuli are referred to here as high autism interest (HAI) stimuli. We note that, although the DSM defines restricted interests to be idiosyncratic and person-specific, and although idiosyncratic restricted interests have been used in ASD studies (Cascio et al., 2014), the use of a standardized set of images allowed for internal validity in that stimuli viewed by different participants and different diagnostic groups did not differ in semantic content or visual features (e.g., luminance, contrast, etc.).

#### Other Non-Social Stimuli

A complimentary set of non-social stimuli that are not commonly the focus of restricted interests in ASD (e.g., clothes and nature) was used as well (images are presented in the Appendix of Dichter et al., 2012a). These stimuli are referred to here as low autism interest (LAI) stimuli.

#### Practice Stimuli

For the task practice session, stimuli consisted of neutral, non-social images that did not overlap with the experimental stimuli.

### Monetary Choice Task (See Figure 1)

An econometric choice task was used to examine choice-based valuation of the three image categories (Watson et al., 2009). This task is structured to mimic the constraints of naturalistic information foraging, requires no explicit rule learning, and has been successfully used in non-human primates to measure the subjective value of social images (Deaner et al., 2005; Watson et al., 2009). Moreover, in humans, this task provides a metric of valuation for social images that departs from explicit ratings of attractiveness (Watson et al., 2010). Participants decided between maximizing a cash payout and sacrificing cash for the opportunity to view images of different categories. On each trial, participants choose between constant and variable value targets. The constant target results in presentation of a phase-scrambled image from one image category and the sound of jingling coins for 500 ms, whereas the variable target results in presentation of an image from the same category and the sound of jingling coins for 300, 500, or 700 ms, which varied across blocks. Participants were instructed that the duration of the jingling coin sound corresponded to the amount of money earned on each trial, with longer sounds corresponding to larger payoffs. Trials were presented in 12-trial blocks of variable reward duration, presented within three superblocks of image type (i.e., three 36 trial “superblocks”—social, HAI, and LAI—each of which contained three 12-trial blocks—one each of 300, 500, and 700 ms variable reward lengths). The order of presentation of superblocks (social, HAI, and LAI) and reward blocks were counterbalanced across participants. Changes in both reward lengths and image category were not explicitly signaled, requiring participants to periodically sample each option in order to reveal task contingency changes. After each superblock, cumulative earnings and earnings for the previous superblock were presented, and participants were compensated a base rate plus the amount earned during the task.

## Results

Data were analyzed using a mixed effects logistic regression using the lme4 package in R. Individual choices (image vs. phase-scrambled) were the dependent measure in the model, and image category, diagnostic group, and cash payouts were fixed effects, and participant was a random effect.

Figure 2 illustrates the percentage choice for images of each category (vs. scrambled images) for ASD and control participants for responses during the superblocks presenting LAI (left), HAI (middle), and social (right) images. Overall, choices were strongly influenced by the effect of diagnostic group (control vs. ASD), as a model including this factor significantly (*p* < 0.001) outperformed a model that did not. Across stimulus categories, both control and ASD participants valued image more when paired with an increasingly large cash reward payout, indicated by the linear relationship between choice behavior and cash outcome, *p* = 0.03. This pattern was particularly true for LAI images (see Figure 2A), for which there was a cash outcome × image category interaction, *p* < 0.0001, indicating that both groups chose to view LAI images more frequently for increasingly large cash payouts. Specifically, both groups chose to view the LAI images about 50% of the time when paired with low payout, about 60% of the time when paired with medium payout, and about 68–70% of the time when paired with high payout. However, diagnostic group did not differentiate valuation for LAI images *p* > 0.79.

Our primary finding was that diagnostic groups differed in image valuation for HAI images (see the middle panel of Figure 2). Whereas control participants were relatively indifferent to cash payouts and chose to see HAI images about 55% of the time across payout conditions, the ASD group clearly preferred the HAI images, choosing them 62–70% of the time (cash outcome × by diagnostic group interaction *p* < 0.001), indicating a willingness in the ASD group to receive less cash payout to view the HAI images.

**FIGURE 1:**
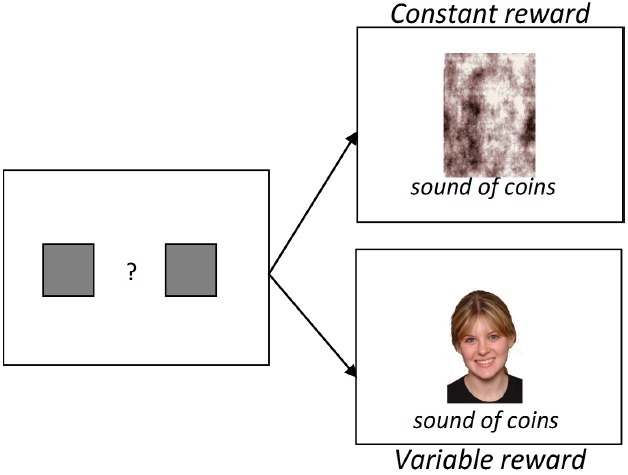
**During the monetary choice task, participants choose, via button press, between (1) a phase-scrambled image paired with a constant value reward (e.g., sound of coins for 500 ms) and (2) a social, HAI, or LAI image paired with a variable reward (sound of coins for 300, 500, or 700 ms).** The duration of the coin sounds corresponded to the amount of money earned.

Contrary to predictions, as can be seen in the right panel of Figure 1, groups did not differ in their willingness to forgo cash payouts to view social images (*p* = 0.29). Both groups chose to view to social images 51–55% of the time when paired with low payout, 57–62% of the time when paired with medium payout, and 58–61% of the time when paired with high payout.

**FIGURE 2:**
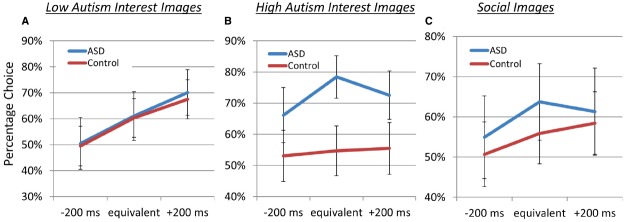
**Percentage choice for images of each category (vs. scrambled images) for ASD and control participants.** “Equivalent” denoted that choosing to see the image resulted in the sound of coins jingling for 500 ms, whereas “–200 ms” and “+200 ms” denote that choosing to see the image would results in the sound of coins jingling for 300 or 700 ms, respectively. Choosing to see the phase-scrambled image always resulted in the sound of coins jingling for 500 ms, and participants were compensated a base rate plus the amount earned during the task. Depicted are responses during the superblock presenting low autism interest **(A)**, high autism interest **(B)**, and social **(C)** images.

There was a significant effect of varying the cash payout, with a positive linear relationship between increasing payouts and the probability of choosing the associated option (*p* = 0.03). Notably, this positive relationship between choice probability and payout amount was not different between the control and ASD groups (*p* > 0.23). This equivalent response to cash payout is most obvious for the LAI image category (Figure 2).

To evaluate whether valuation of image categories predicted the severity of ASD symptoms within the ASD group, correlations between total and subscale scores on the Social Responsiveness Scale (Constantino and Gruber, 2002) and the Repetitive Behavior Scale-Revised (Bodfish et al., 1999) and percentage choices for each category were evaluated. No significant correlations were found, even at uncorrected significance thresholds.

## Discussion

We found that children and adolescents with ASD were willing to forgo cash payouts in order to view non-social images related to restricted interests in ASD but found no group differences in willingness to forgo cash payouts in order to view other non-social images or social images. These are the first behavioral econometric data to illustrate that images related to restricted interests are more valuable for children and adolescents with ASD than their TD counterparts.

Our findings are consistent with studies that find altered behavior or neural activity during motivated tasks involving non-social stimuli (Schmitz et al., 2008; Scott-Van Zeeland et al., 2010; Kohls et al., 2011, 2012; Dichter et al., 2012a; Ewing et al., 2013; Pankert et al., 2014), but not those that find altered responses to social rewards (Geurts et al., 2008; Demurie et al., 2011; Delmonte et al., 2012; Lin et al., 2012; Stavropoulos and Carver, 2014). The finding of group differences in econometric choices for non-social images related to restricted interests highlight that the social motivation theory of ASD may be too constrained, and that altered motivational processes in ASD may extend to restricted and repetitive behaviors in ASD as well (e.g., Kohls et al., 2014). These findings are consistent with prior reports that the restricted interest images used in this study elicit higher subjective ratings of pleasure from individuals with ASD (Sasson et al., 2012) as well as functional neuroimaging reports that restricted interests stimuli elicit greater activation in canonical reward processing brain regions (Dichter et al., 2012a; Cascio et al., 2014).

The lack of group differences in response to social images was contrary to a prediction derived from the social motivation theory of ASD, which posits that social stimuli may be less motivationally salient for individuals with ASD (Chevallier et al., 2012b). It may be the case that, whereas static images related to restricted interests in ASD may have been an adequate laboratory-based “press” to elicit altered motivational responses, static social stimuli may not have been an adequate proxy for dynamic social stimuli that would be encountered in real-word contexts, a phenomenon that has been reported in other studies (Weisberg et al., 2014; Chevallier et al., 2015). Indeed, recent conceptual models of ASD that highlight deficits in ASD in the ability to process unexpected or unpredictable events may depend on the use of dynamic stimuli to elicit social impairments in ASD in the laboratory setting (Sinha et al., 2014; Van de Cruys et al., 2014).

The present study has a number of interpretive cautions. First, the size of the ASD sample was relatively small, which potentially reduced statistical power, particularly for analyses testing for relations between the severity of core ASD symptoms and individual differences in behavioral econometric valuations. Additionally, a number of studies have documented anomalous behavioral and neural responses to monetary incentives in ASD (Schmitz et al., 2008; Scott-Van Zeeland et al., 2010; Kohls et al., 2011, 2012; Dichter et al., 2012a,b; Lin et al., 2012; Richey et al., 2014), and thus it is not clear that monetary incentives in the present study were processed equivalently by children and adolescents with and without ASD. Indeed, the value of money may be contingent on a social exchange with another person, thereby providing a social overlay to monetary rewards for individuals with ASD. Despite these caveats, we note that, in our study, choices of both ASD and TD participants were biased toward options associated with the larger cash payout, indicating a sensitivity to monetary incentives.

Despite these interpretive cautions, our central finding that children and adolescents with ASD were willing to receive smaller cash payouts to view images related to restricted interests highlights the motivational relevance of these stimuli for individuals with ASD. This finding also has implications for ASD interventions. Many intensive, early behavioral interventions for ASD use rewards to reinforce appropriate social behaviors. For example, the Early Start Denver model relies on reinforcers for social interactions, such as parental praise and shared engagement with joint activities (Dawson et al., 2010). Likewise, applied behavior analysis (ABA) teaches new skills through the use of a predictable delivery schedule of explicit rewards (Koegel et al., 1999; Jensen and Sinclair, 2002). The present findings highlight the potential utility of leveraging restricted interests as potent rewards themselves in these contexts, given that they have high implicit value for children with ASD. This idea has been suggested previously (Charlop and Haymes, 1996; Boyd et al., 2007), but the present findings provide empirical support for the implicit motivational salience of restricted interests stimuli for children with ASD.

The present study did not have a large enough sample to explore developmental influences on valuation of social and non-social stimuli in ASD, and future research with larger samples will be needed to evaluate the implicit value of social and non-social stimuli earlier in childhood and on into adulthood in ASD. Additionally, given that restricted interests in ASD are, by definition, idiosyncratic, future research with images of person-specific restricted interests may yield more pronounced effects. Likewise, as previously mentioned, dynamic social stimuli may be needed to elicit differences in social valuation in ASD. Despite these limitations, the present study represents to first behavioral econometrics data to estimate the implicit reward value of social and non-social stimuli in children with ASD and highlights the utility of behavioral econometric approaches to studying motivational processes in ASD.

### Conflict of Interest Statement

The authors declare that the research was conducted in the absence of any commercial or financial relationships that could be construed as a potential conflict of interest.
